# Distribution of *Chlamydia trachomatis ompA* genotypes and its association with abnormal cervical cytology among women of reproductive age in Shenzhen, China

**DOI:** 10.3389/fpubh.2022.1036264

**Published:** 2022-10-31

**Authors:** Lan-lan Liu, Si Sun, Li Zhang, Qiu-hong Wu, Li-shan Tian, Bo Li, Xiang-sheng Chen, Zhen-zhou Luo

**Affiliations:** ^1^Shenzhen Nanshan Center for Chronic Disease Control, Shenzhen, China; ^2^National Center for STD Control, Chinese Academy of Medical Sciences and Peking Union Medical College Institute of Dermatology, Nanjing, China

**Keywords:** cervical intraepithelial lesion, *Chlamydia trachomatis*, genotyping, human papillomavirus, women of reproductive age

## Abstract

**Background:**

Many studies have focused on the distribution and specific clinical symptoms caused by *Chlamydia trachomatis*. Still, relatively few studies have focused on the associations between *Chlamydia trachomatis* genotypes and cervical intraepithelial lesions.

**Objectives:**

This study was conducted to determine the distribution of *Chlamydia trachomatis* genotypes and its associations with cervical intraepithelial lesions among women of reproductive age. The presence of other STIs coinfection was also evaluated.

**Method:**

375 *Chlamydia trachomatis* positive cervical swabs collected from women of reproductive age were analyzed though molecular assay. Multivariate logistic regression analyses (covariates include contraception, gravidity (≥1), abnormal vaginal discharge, adverse pregnancy outcomes, reproductive tract symptoms and abnormal cervical cytology) were performed to evaluate the associations between *Chlamydia trachomatis* genotypes and cervical intraepithelial lesions and genital clinical symptoms.

**Results:**

Among 375 *Chlamydia trachomatis* positive cervical swabs, the prevalence of coinfection with *Neisseria gonorrhoeae, Candida albicans, Trichomonas vaginitis, Vulvovaginal candidiasis*, and HPV were 0.8%, 2.7%, 2.4%, 10.1% and 15.5%, respectively. 306 were genotyped successfully, and nine genotypes were identified. The most common genovar was E (25.16%, 77/306), followed by J (22.55%, 69/306), F (17%, 52/306), D (14.4%, 44/306), K (7.2%, 22/306), G (6.9%, 21/306), H (5.2%, 16/306), B (1.0%, 3/306), Ia (0.7%, 2/306). Genotype H was associated with abnormal cervical cytology [*p* = 0.006, aOR = 8.16 (1.86–36.6)]. However, this study observed no association between *Chlamydia trachomatis* genotypes and any genital clinical symptoms.

**Conclusions:**

*Chlamydia trachomatis* genotype H may be a high risk factor for cervical intraepithelial lesions, which is useful for treatment and management measures for patients with cervical intraepithelial lesions.

## Introduction

*Chlamydia trachomatis* (*C.trachomatis*), an obligate intracellular bacterium, remains the most frequent causative agent of sexually transmitted infections (STIs) worldwide, with a global incidence of 38 per 1,000 women and 33 per 1,000 men (1). 105 sentinel surveillance sites in China reported that *C.trachomatis* incidence increased from 35.8 per 100,000 in 2011 to 37.1 per 100,000 in 2015. Men who have sex with men (MSM) and female sex workers (FSW) have a higher risk of *C.trachomatis* infection, accounting for 6.5% and 17.3% of infections, respectively (2, 3). The true *C.trachomatis* prevalence may be underestimated because most cases are asymptomatic (about 70% of women and 50% of men don't have any clinical symptoms) (4).

Based on the major outer membrane protein (MOMP) encoded by the *ompA* gene, 19 *C.trachomatis* serovars have been classified into three clusters: genotype A-C (predominantly related to trachoma), genotype D-K (associated with urogenital infections), genotype L_1_-L_3_ (causing lymphogranuloma venereum) (5). Left untreated, *C.trachomatis* can lead to severe consequences, such as cervicitis, cervical ectopy and chronic pelvic inflammation in females, urethritis in males, and neonatal conjunctivitis in newborns (6).

Cervical cancer (CC) is the fourth most common cancer amongst women worldwide (7). The major risk factors associated with CC development include high-risk human papilloma virus (hrHPV) infection, age, smoking, childbirth, use of oral contraception, and diet (8–13). The association between certain hrHPV of HPV and cervical cancer is well established (14). However, previous studies show that only a few HPV infection cases will develop into CC (15). That means HPV alone is not sufficient for the development of CC.

CC arises from normal cervical epithelium through the progressive development of low grade and high grade cervical intraepithelial lesions (CINs) (16). And *C.trachomatis* is considered as a risk factor for abnormal cervical cytology in previous researches (17–19). However, it is unclear that which are high-risk and which are low-risk for CINs about 19 *C.trachomatis* genotypes, which may facilitate the prevention and treatment of *C.trachomatis* infection. Unfortunately, only a few studies have been conducted to evaluate the association between *C.trachomatis* genotypes and CINs (20). Thus, this study aims to determine the distribution of *Chlamydia trachomatis* genotypes and its associations with CINs among women of reproductive age.

## Materials and methods

### Study design

From March to August 2017, we recruited participants from 9,249 women who met eligibility criteria and provided informed consent in our previous study (21), and all of the women signed an informed consent to this study. Women who met any of the following exclusion criteria were not enrolled: pregnancy, without a history of sexual activity, sexual intercourse 3 days ago, menstrual period, previous hysterectomy, vaginal bleeding, vaginal douching or using a vaginal suppository, currently suffering from gynecological inflammation. The inclusion criteria were the same as inclusion criteria in our previous study (21): being a female resident, aged 20–60 years and living locally in Shenzhen city Nanshan District during the past 3 months. All participants signed informed consent and were interviewed using a structured questionnaire to collect socio-demographic and clinical information before enrollment. All participants voluntarily agreed to provide a self-administrated 3–5 mL first-catch urine specimen (Chlamydia trachomatis and Neisseria gonorrhoeae tests), a cervical swab (HPV tests), two vaginal swabs (gynecological examinations), and an exfoliated cervical cells specimen (liquid-based cervical cytology test).

### Positive endocervical swabs samples

Out of the 9,249 women who met eligibility criteria and signed an informed consent, 9,090 (98.3%) women's specimens were successfully tested, and 375(4.13%, 375/9,090) were *C.trachomatis* positive.

375 positive endocervical swabs samples were used for *C.trachomatis* genotyping to evaluate association with CINs. More detailed study methods and epidemiological information about the study are available in a previously published article (21, 22).

### C.trachomatis and neisseria gonorrhoeae DNA test

The method and procedures of *C.trachomatis* and *Neisseria gonorrhoeae* DNA test were described in our previous study (21).

### *C.trachomatis* DNA extraction, *OmpA* PCR amplification, sequencing and genotyping

The positive endocervical swabs were each eluted with 1 ml sterile water and vortexed. Researchers took 200 μl of eluant from the samples for DNA extraction. DNAs were extracted by using QIAamp^®^ cador^®^ Pathogen Mini Kit (Qiagen, Hilden, Germany), according to the manufacturer's instructions. The remaining eluate was stored at −80°C.

Molecular genotyping of *C.trachomatis* was performed by *ompA* gene; the detailed information about *ompA* primers for the current nested PCR system is available at the Uppsala University *C.trachomatis* MLST database (http://mlstdb.bmc.uu.se). The nested PCR cycling protocol methods was previously described (23). The secondary PCR amplifications were purified and sent to be sequenced by HYK-High-throughput Biotechnology Institute (Shenzhen, China). All PCR amplifications were sequenced bidirectionally.

### HPV DNA testing and genotype

HPV DNA testing and genotyping (14 HR-HPV including genotype 16, 18, 31, 33, 35, 39, 45, 51, 52, 56, 58, 59, 66, 68 and 2 low-risk HPV including genotype 6 and 11) were conducted by the Beijing Genomics Institute according to operating instructions (24).

### ThinPrep cytological test (TCT)

The cytobrush containing cervical exfoliated cells was collected and stained, and then fixed in TCT cytological solution for 15 min. TCT was performed by a SurePath liquid-based Pap test (BD, United States), according to the manufacturer's instructions. Cervical cytology test results were classified by the Bethesda System (TBS; 2001) criteria as follows: negative for intraepithelial lesion or malignancy (NILM); atypical glandular cells (AGC); squamous intraepithelial lesions (SIL) of low (LSIL) or high (HSIL) grade; atypical squamous cells (ASC) of undetermined significance (ASC-US) or not possible exclude HSIL (ASC-H); and squamous cell carcinoma (SCC) (25). In this study, cervical cytology was dichotomized into normal (NILM) and abnormal (≥ASC). More detailed information is available in our previous study (22).

### Vaginal cleanliness evaluation

Vaginal smears on clean glass slides were observed under microscope at the hospital laboratory. The evaluation criteria of vaginal cleanliness was divided into 4 grades by the relative abundance of *Lactobacillus* spp., vaginal epithelial cells, pus cells and white blood cells per high power field (26). Grade I and grade II indicate normal vaginal cleanliness, whereas grade III and grade IV are abnormal, indicating the presence of inflammation or infection (22).

### Gynecological examination

Vaginal secretions swab specimens were collected by two skilled gynecologists and were rolled on to a glass slide for Gram staining immediately. Vaginal cleanliness, detection of hyphae and spores of Candidiasis and clue cells were confirmed by Gram staining of vaginal secretions. Trichomonas Vaginalis was diagnosed by microscopic examination of wet mounts immediately once the vaginal secretions swab collected. Amine test, pH of vaginal secretions and leukocytes were further confirmed within 15 min. The diagnosis of bacterial vaginosis was based on Amsel's criteria (27), which was widely adopted to the clinical diagnosis of bacterial vaginosis. A positive diagnosis of bacterial vaginosis was made once three of the four following signs are present: the presence of clue cells, an adherent and homogenous grayish-white vaginal discharge, a vaginal pH exceeding a value of 4.5, a fishy or amine odor after the addition of a 10% potassium hydroxide solution. The method and procedures of gynecological examination were described in our previous study (22).

### Statistical analyses

Raw data collection and statistical analysis were performed by Microsoft Excel 2016 and SPSS Statistics version.20.0 (SPSS Inc., Chicago, IL, United States), respectively. Abnormal cytology group (≥ASC-US was defined as women who had a diagnosis of the following cytology findings: ASC-US, ASC-H, LSIL, HSIL or AGC). *C.trachomatis* genotypes were divided into two group, genotype B group and non-B genotypes group, genotype D group and non-D genotypes group, genotype E group and non-E genotypes group, and so on. The chi-square test (χ^2^), or two-sided Fisher exact test for 2 × 2 contingency table was used to evaluate the associations between different *C.trachomatis* genotypes, sociodemographic characteristics, reproductive history, sexual behavior, and urogenital symptoms. Variables with a significance level of *p* < 0.2 were enrolled in the multivariate logistic regression model adjusted by potential confounders. Crude odds ratios (OR), adjusted odds ratio (AOR) and corresponding 95% CIs were calculated. A *p* < 0.05 was considered significant.

### Ethics statement

The study was approved and overseen by Ethical Committee of the Shenzhen Nanshan Center for Chronic Disease Control (Approved No. LL20170017).

## Results

### Population characteristics

Socio-demographic and clinical information in patients with *C.trachomatis* infection were shown in Table 1. A total of 69.1% of C.trachomatis positive women were >35 years old; 73.9% of *C.trachomatis* positive women were middle and low-income. More than half of women were unemployed (63.5%). 94.1% of women were married, and 80.3% reported using condoms or other contraceptive methods, including oral contraceptive use and intrauterine devices; 54.7% had no reproductive tract symptoms.

**Table 1:** Sociodemographic and clinical information in patients with *C.trachomatis* infection.

**Variables**		**Number(*n* = 375)**	**%**
Age	≤ 35	116	30.9
	>35	259	69.1
Income	< 4,000 RMB	220	58.7
	4,000–8,000 RMB	132	35.2
	>8,000 RMB	23	6.1
Employment status	Unemployed	238	63.5
	Employed	137	36.5
Registered residence	Local	146	38.9
	Nonlocal	229	61.1
Marital status	Married	353	94.1
	Unmarried^a^	22	5.9
Ethnicity	Han	353	94.1
	Minority	22	5.9
Oral contraceptive	No	301	80.3
	Yes	74	19.7
Condom use	No	125	33.3
	Yes	250	66.7
APOs^b^	No	285	76.0
	Yes	90	24.0
Delivery modes	Vaginal delivery	88	23.5
	Cesarean delivery	267	71.2
	Never had children	20	5.3
Vaginal discharge	No	205	54.7
	Yes	170	45.3
Gravidity	No	13	3.5
	Yes	362	96.5
Previous *C.trachomatis* test	No	340	90.7
	Yes	22	5.9

a Unmarried include single, divorced.

b APOs: adverse pregnancy outcomes, refers to spontaneous abortion, stillbirth, preterm labor, low birth weight, infertility, infant death, ectopic pregnancy.

A total of 24% of women had adverse pregnancy outcomes. Cesarean delivery accounted for 71.2%. 90.7% had never had a *C.trachomatis* test previously.

### C.trachomatis coinfection with other STIs and Liquid-based cytology test results

As shown in Table 2, the most prevalent coinfection pathogen was HPV, which accounted for 15.47%, particularly high-risk HPV (96.55%, 56/58), followed by *vulvovaginal candidiasis* (10.13%), *candida albicans* (2.67%), *trichomonas vaginitis* (2.4%), and NG (0.8%).

**Table 2:** *C.trachomatis* coinfection with other STIs.

**Coinfecting pathogens**	**Test results**	**No**.	Prevalence (%, 95% CIs)^*^
NG^a^	Negative	372	
	Positive	3	0.8 (1–1.7)
*Candida albicans*	Negative	365	
	Positive	10	2.67 (1.04–4.3)
*Trichomonas vaginitis*	Negative	366	
	Positive	9	2.4 (0.85–3.95)
*Vulvovaginal candidiasis*	Negative	337	
	Positive	38	10.13 (7.08–13.19)
HPV	Negative	317	
	Positive	58	15.47 (11.81–19.13)
Multiple HPV infection	No	370	
	Yes	5	1.33 (0.17–2.49)
HR-HPV^b^	Negative	319	
	Positive	56	14.93 (11.33–18.54)
Liquid-based cytology test	Normal	346	
	Abnormal	29	7.73 (5.03–10.44)

a NG refers to neisseria gonorrhoeae.

b HR-HPV refers to high-risk HPV.

* 95% CIs, 95% confidence intervals, *p* < 0.05 was considered significant.

### The distribution of C.trachomatis genotypes by cytology status

As shown in Table 3, among 375 *C.trachomatis* positive cervical swabs, 306 were genotyped successfully, and 69 samples containing less *C.trachomatis* DNA were insufficient for genotyping. Out of 306 samples, nine genotypes were identified. The most common genotype was E (25.16%, 77/306), followed by J (22.55%, 69/306), F (17%, 52/306), D (14.4%, 44/306), K (7.2%, 22/306), G (6.9%, 21/306), H (5.2%, 16/306), B (1.0%, 3/306), and Ia (0.7%, 2/306).

**Table 3:** The prevalence of *C.trachomatis* genotypes by cytology status.

***C.trachomatis* genotypes**	**Overall (*n* = 306)**	**NILM^a^ (*n* = 284)**	**Abnormal cervical cytology (*n* = 22)**	***p***	OR (95% CIs)^*^
	***n* (%)**	***n* (%)**	**n (%)**		
B^b^	3 (0.98)	3 (1.06)	0	-	-
D^c^	44 (14.38)	43 (15.14)	1 (4.55)	0.294	0.27 (0.04–2.04)
E	77 (25.16)	71 (25)	6 (27.27)	0.813	1.13 (0.42–2.99)
F	52 (16.99)	49 (17.25)	3 (13.64)	0.888	0.76 (0.22–2.66)
G	21 (6.86)	19 (6.69)	2 (9.09)	1	1.4 (0.3–6.42)
H	16 (5.23)	11 (3.87)	5 (22.73)	0.001	7.3 (2.28–23.41)
I	2 (0.65)	2 (0.7)	0	-	-
J	69 (22.55)	64 (22.54)	5 (22.73)		1.01 (0.36–2.85)
K	22 (7.19)	22 (7.75)	0	-	-

a NILM refers to negative for intraepithelial lesion or malignancy, means normal cervical cytology.

b ,

c genotype B, compare to non-genotype B (the rest genotypes, include D, E, F, G, H, Ia, J, and K); genotype D, compare to non-genotype D (the rest genotypes, include B, E, F, G, H, Ia, J, and K). The rest genotypes can be done in the same manner.

* 95%CIs, 95% confidence intervals, *p* < 0.05 was considered significant.

22 (7.19%) samples had abnormal cervical cytology; The remaining were negative for intraepithelial lesion or malignancy (NILM) (92.81%, 284/306). We found a positive association between *C.trachomatis* genotype H infection and abnormal cervical cytology [*p* = 0.001, OR = 7.3 (95% CI: 2.28–23.41)].

### Associations between C.trachomatis genotypes and clinical characteristics

As shown in Table 4, after adjusting for potential confounders by multivariate logistic regression analysis, we found that no associations were observed between different *C.trachomatis* genotypes and gravidity, vaginal cleanliness, adverse pregnancy outcomes or reproductive tract symptoms. However, the association of genotype K with contraception, including condom use, oral contraceptive use, and intrauterine devices was observed (*p* = 0.03, aOR = 0.36, 95% CI: 0.14–0.93). Genotype D was associated with gravidity (*p* = 0.02, aOR = 0.2, 95% CI: 0.05–0.75). Most importantly, compared to other CT genotypes, there was a significant association between genotype H and abnormal cervical cytology (*p* = 0.006, aOR = 8.16, 95% CI: 1.83–36.6).

**Table 4:** Associations between *C.trachomatis* genotypes and clinical characteristics.

**Genotypes**	***n* (%, *n* = 306)**	**Contraception**			**Gravidity (**≥**1)**			**Abnormal vaginal discharge**		
		***n* (%)**	***p***	**aOR^a^ (95% CI)**	***n* (%)**	***p***	**aOR (95% CI)**	***n* (%)**	***p***	aOR (95% CI)^*^
B^b^	3 (1.0)	1 (33.3)	0.99	-	3 (1.0)	1	-	1 (33.3)	0.5	0.43 (0.04–4.93)
D	44 (14.4)	32 (72.7)	0.79	0.89 (0.39–2.01)	39 (88.6)	0.02	0.2 (0.05–0.75)	27 (61.4)	0.19	1.56 (0.81–3.03)
E	77 (25.2)	48 (54.5)	0.18	1.65 (0.80–3.39)	76 (98.7)	0.22	3.68 (0.46–29.6)	36 (46.8)	0.3	0.76 (0.45–1.28)
F	52 (17.0)	35 (67.3)	0.55	1.27 (0.58–2.81)	51 (98.1)	0.64	1.65 (0.20–13.5)	25 (48.1)	0.52	0.82 (0.45–1.50)
G	21 (6.9)	13 (61.9)	0.26	2.38 (0.53–10.66)	21 (1.0)	1	-	12 (57.1)	0.64	1.24 (0.50–3.04)
H	16 (5.2)	13 (81.3)	0.94	1.05 (0.28–3.92)	16 (1.0)	1	-	9 (56.3)	0.64	1.28 (0.45–3.64)
Ia	2 (0.7)	1 (50.0)	0.99	-	2 (1.0)	1	-	1 (50.0)	0.91	1.18 (0.07–19.3)
J	69 (22.5)	41 (59.4)	0.14	0.61 (0.31–1.17)	66 (95.7)	0.93	0.94 (0.24–3.71)	37 (53.6)	0.77	1.08 (0.63–1.86)
K	22 (7.2)	19 (86.3)	0.03	0.36 (0.14–0.93)	20 (90.9)	0.16	0.31 (0.06–1.59)	11 (50.0)	0.85	0.92 (0.38–2.20)
**Genotypes**	***n*** **(%**, ***n*** = **306)**	**Adverse pregnancy outcomes**			**Reproductive tract symptoms**			**Abnormal cervical cytology** ^c^		
		***n*** **(%)**	***p***	**aOR (95% CI)**	***n*** **(%)**	***p***	**aOR (95% CI)**	***n*** **(%)**	***p***	**aOR (95% CI)**
B	3 (1.0)	0	-	-	1 (33.3)	0.87	1.22 (0.11–13.9)	0	-	-
D	44 (14.4)	7 (15.9)	0.14	0.52 (0.22–1.24)	27 (61.4)	0.17	1.61 (0.82–3.15)	1 (2.30)	0.27	0.30 (0.04–2.57)
E	77 (25.2)	21 (27.3)	0.41	1.28 (0.71–2.33)	22 (28.6)	0.81	0.93 (0.53–1.65)	6 (7.80)	0.73	1.21 (0.42–3.50)
F	52 (17.0)	9 (17.3)	0.32	0.63 (0.31–1.47)	15 (28.8)	0.95	1.02 (0.53–1.98)	3 (5.80)	0.43	0.58 (0.15–2.28)
G	21 (6.90)	7 (33.3)	0.25	1.77 (0.68–4.60)	6 (28.6)	0.92	0.95 (0.36–2.56)	2 (9.50)	0.99	1.01 (0.19–5.37)
H	16 (5.20)	4 (25.0)	0.76	1.2 (0.37–3.90)	3 (18.8)	0.36	0.55 (0.15–1.99)	5 (31.3)	0.006	8.16 (1.83–36.6)
Ia	2 (0.70)	2 (100)	1	-	1 (50.0)	0.59	2.14 (0.13–30.5)	0	-	-
J	69 (22.5)	17 (24.6)	0.87	1.06 (0.56–1.98)	20 (29.0)	0.87	0.95 (0.52–1.72)	5 (7.20)	0.81	1.15 (0.37–3.55)
K	22 (7.20)	5 (22.7)	0.98	1.01 (0.36–2.87)	5 (22.7)	0.52	0.71 (0.25–1.99)	0	-	-

a aOR: refers to adjusted odds ratio.

b B genotype, compared to non-genotype B (the rest genotypes, include D, E, F, G, H, Ia, J, and K). The rest genotypes can be done in the same manner.

c Abnormal cervical cytology refers to ASC (atypical squamous cells), SIL (squamous intraepithelial lesions), ASC-US (atypical squamous cells of undetermined significance), and normal cervical cytology refers to NILM.

* 95%CIs, 95% confidence intervals, *p* < 0.05 was considered significant.

## Discussion

In this study, 375 *C.trachomatis* positive cervical swabs were collected from a population-based cross-sectional survey with a relatively larger sample size (*n* = 9,090) (28). This study extended the existing literature by distribution of *C.trachomatis ompA* genotypes and its associations with abnormal cervical cytology.

In this study, we found that most participants had no *C.trachomatis* test previously, which indicated that they were unaware of the cervical lesions that *C.trachomatis* cause. And almost half reported that they had no vaginal discharge, which aligns with the fact that many infections are asymptomatic, thus delaying diagnosis.

As for coinfection, the most prevalent coinfection pathogen was HPV, especially high-risk HPV. The prevalence of coinfection with NG was relatively low in our study.

In this study, 306 samples were genotyped successfully and 69 samples containing less *C.trachomatis* DNA were insufficient for genotyping. The failure rate of *C.trachomatis ompA* gene sequencing in this study (18.4%, 69/375) was higher than in a study conducted in mainland China (5.83%, 14/240), but lower than other studies (Taiwan, 29.6%, 43/145; mainland China, 20.2%, 33/163) (29–31). The difference may be due to clinical samples (cervical swabs and urine) and different sequence methods performed in different studies.

Out of 306 successfully genotyped samples, nine different *C.trachomatis* genotypes from B and D-K were detected. The results in this study were diverse when compared with other studies (31, 32). We speculate that the specific location of the study, Shenzhen, may be linked to the diversity. Because Shenzhen is a modern city with rapid economic development and large unregistered, young, floating migrant population. It has been reported that *C.trachomatis* is the most common STI mainly affecting young individuals (1).Thus, it is reasonable that a diverse array of genotypes existed in our study. And similar to other studies, genotypes E, F, J, and D were the most prevalent in Guangdong (1).

Among nine *C.trachomatis* genotypes, E and J were the predominant genotypes in this study, which is slightly different when compared with two studies carried out in mainland China where F and E, D, and E genotypes were the most common, respectively (28, 31). Genotype E was one of the stable genotypes among the general population despite the existing difference, while G and D were identified as the most prevalent genotypes among MSM (33). Lymphogranuloma venereum genotype L1-L3 was absent in our study, but was also recognized in the MSM (34).

Interestingly, genotype B was detected in cervical swabs in our study, it was found associated with trachoma and neonatal conjunctivitis in other studies previously (5, 32, 35). This results indicated that genotype B may lead to multiple anatomical sites infection, not only caused eye infection, but also reproductive tract infection. To our knowledge, this was the first time that genotype B was found among women in Shenzhen, excluding a previous study conducted in MSM (33).

We observed that clinical manifestations including abnormal vaginal discharge, were not associated with any genotypes. These results are in accordance with some research, but different from others (1, 32, 36). Previous studies have shown that genotype K was associated with abnormal vaginal discharge, and genotype G was related to low abdominal pain (30, 37). Although the association between parity and STIs has been demonstrated, the association between parity and genotypes was not found in this study (17, 38).

The rate of abnormal cervical cytology in this study (7.2%) was higher than in a previous study (2.8%) (39). However, in terms of the relationship between *C.trachomatis* positive and cervical precancerous lesions, our findings varied with the previous literature (17, 18). A case-control study and a meta-analysis showed that *C.trachomatis* infection was a higher risk factor of CC, while other studies found no association between the *C.trachomatis* and CC or abnormal cervical cytology (20, 39, 40). Our result was similar to the latter.

In present study, genotype E and J are the most frequent genotypes, but these two was not significantly related to the abnormal cervical cytology, suggesting that genotype E may be not the pathogenic factor for abnormal cervical cytology. Genotype H was associated with abnormal cervical cytology, indicating that genotype H may be the pathogenic factor for abnormal cervical cytology, which is a little different from the previous study reported by Chen (1). They found that patients with genotype G infection commonly had abnormal cervical cytology (*p* = 0.029, OR = 1.868, 95% CI: 1.124–3.106). However, the association between genotype G and abnormal cervical cytology was not observed in our study (1). Genotypes B, D, G, and I were shown to be related to abnormal cervical cytology in a different case control study, which was also not found in this study (40).

## Limitations

Several limitations of our study should be acknowledged. First, the genotyping method used in our study could not identify strains with multiple genotypes infections, despite being sensitive and specific. Second, the failure rate of *C.trachomatis ompA* gene sequencing in this study (18.4%, 69/375) was a little higher, which may influence the study's conclusion. Third, the sample size (*n* = 375) of this study was small, and samples collected in the study were limited to the Nanshan district, Shenzhen, so the representativeness of genotype distribution may be affected. Further research is needed to enroll a population comprising of both males and females and enlarge the target region.

## Conclusions

In conclusion, *C.trachomatis* genotype H may increase the risk of cervical intraepithelial lesion in terms of cross-sectional epidemiology, which indicates that H is a higher risk *C.trachomatis* genotype. This provides some evidence for clinical diagnosis and treatment of *C.trachomatis*.

## Data availability statement

The original contributions presented in the study are included in the article/supplementary material, further inquiries can be directed to the corresponding author.

## Ethics statement

The study was approved and overseen by Ethical Committee of the Shenzhen Nanshan Center for Chronic Disease Control (Approved No. LL20170017). The patients/participants provided their written informed consent to participate in this study.

## Author contributions

Conceptualization: X-sC, BL, Z-zL, L-lL, SS, LZ, Q-hW, and L-sT. Data curation: L-lL, Q-hW, and LZ. Formal analysis: L-lL, Q-hW, BL, and X-sC. Investigation: L-lL, Q-hW, LZ, and L-sT. Methodology: L-lL, SS, and Q-hW. Project administration and resources: Z-zL, L-lL, and SS. Software: L-lL and SS. Supervision: BL, Z-zL, L-sT, and X-sC. Validation: BL, X-sC, and Z-zL. Visualization: BL and X-sC. Writing—original draft and writing—review & editing: L-lL. All authors contributed to the article and approved the submitted version.

## Funding

This study received financial support from the Shenzhen Nanshan Science and Technology Planning Project (SZ010).

## Conflict of interest

The authors declare that the research was conducted in the absence of any commercial or financial relationships that could be construed as a potential conflict of interest.

## Publisher's note

All claims expressed in this article are solely those of the authors and do not necessarily represent those of their affiliated organizations, or those of the publisher, the editors and the reviewers. Any product that may be evaluated in this article, or claim that may be made by its manufacturer, is not guaranteed or endorsed by the publisher.
